# Lower Pre-Treatment T Cell Activation in Early- and Late-Onset Tuberculosis-Associated Immune Reconstitution Inflammatory Syndrome

**DOI:** 10.1371/journal.pone.0133924

**Published:** 2015-07-24

**Authors:** Odin Goovaerts, Wim Jennes, Marguerite Massinga-Loembé, Pascale Ondoa, Ann Ceulemans, Chris Vereecken, William Worodria, Harriet Mayanja-Kizza, Robert Colebunders, Luc Kestens, TB-IRIS Study Group

**Affiliations:** 1 Department of Biomedical Sciences, Institute of Tropical Medicine, Antwerp, Belgium; 2 Department of Biomedical Sciences, University of Antwerp, Antwerp, Belgium; 3 Centre de Recherches Médicales de Lambaréné (CERMEL), Albert Schweitzer Hospital, Lambarene, Gabon; 4 Institut für Tropenmedizin, Universität Tübingen, Tübingen, Germany; 5 Department of Global Health and Amsterdam Institute for Global Health and Development, Academic Medical Centre, Amsterdam, The Netherlands; 6 Department of Medicine, Mulago Hospital, Kampala, Uganda; 7 Infectious Diseases Institute, Makerere University College of Health Sciences, Kampala, Uganda; 8 Infectious Diseases Network for Treatment and Research in Africa (INTERACT), Kampala, Uganda; 9 Department of Clinical Sciences, Institute of Tropical Medicine, Antwerp, Belgium; 10 Epidemiology and Social Medicine, University of Antwerp, Antwerp, Belgium; George Washington University, UNITED STATES

## Abstract

**Background:**

Tuberculosis-associated immune reconstitution inflammatory syndrome (TB-IRIS) is an inflammatory complication in HIV-TB co-infected patients receiving antiretroviral therapy (ART). The role of disturbed T cell reconstitution in TB-IRIS is not well understood. We investigated T cell activation and maturation profiles in patients who developed TB-IRIS at different intervals during ART.

**Methods:**

Twenty-two HIV-TB patients who developed early-onset TB-IRIS and 10 who developed late-onset TB-IRIS were matched for age, sex and CD4 count to equal numbers of HIV-TB patients who did not develop TB-IRIS. Flow cytometry analysis was performed on fresh blood, drawn before and after ART initiation and during TB-IRIS events. T cell activation and maturation was measured on CD4+ and CD8+ T cells using CD45RO, CD38, HLA-DR, CCR7 and CD27 antibodies.

**Results:**

CD8+ T cell activation before ART was decreased in both early-onset (77% vs. 82%, p = 0.014) and late-onset (71% vs. 83%, p = 0.012) TB-IRIS patients compared to non-IRIS controls. After ART initiation, the observed differences in T cell activation disappeared. During late-onset, but not early-onset TB-IRIS, we observed a skewing from memory to terminal effector CD4+ and CD8+ T cell populations (p≤0.028).

**Conclusion:**

Our data provide evidence of reduced CD8+ T cell activation before ART as a common predisposing factor of early- and late-onset TB-IRIS. The occurrence of TB-IRIS itself was not marked by an over-activated CD8+ T cell compartment. Late- but not early-onset TB-IRIS was characterized by a more terminally differentiated T cell phenotype.

## Introduction

Paradoxical tuberculosis-associated immune reconstitution inflammatory syndrome (TB-IRIS) is a complication that arises during successful antiretroviral therapy (ART) in HIV-tuberculosis (TB) co-infected patients receiving TB-treatment [1]. TB-IRIS presents in up to 25% of HIV-TB patients as worsening symptoms of TB during ART, despite a favourable response to TB-treatment (hence the name “paradoxical TB-IRIS”) [2]. The syndrome poses a significant diagnostic challenge to physicians and it may require hospitalisation or additional therapy [3,4]. In the majority of patients, TB-IRIS occurs within the first few weeks of ART (early-onset TB-IRIS) [5]. Nevertheless, about 15% of TB-IRIS cases develop later than 3 months and even up to 4 years after starting ART [6,7]. This heterogeneity in time between ART initiation and TB-IRIS contributes significantly to the diagnostic confusion that is already surrounding the syndrome and it is unknown which common and differentiating factors drive these early and late presentations of the disease.

Although the pathogenesis of TB-IRIS is not well understood, the idea that IRIS involves an atypical restoration of pathogen-specific immune responses during ART has gained acceptance [1,8,9]. Known risk factors of TB-IRIS include a high TB-antigen burden and a short interval between initiation of TB treatment and ART. The strongest predictor for developing TB-IRIS, however, is a low CD4+ T cell count prior to ART initiation [10,11]. Low CD4 counts in progressive HIV infection are typically associated with high levels of T cell activation [12–16], which may persist during ART. Persistent T cell activation during successful ART, as measured by expression of CD38 and HLA-DR, suggests an incomplete recovery of the immune system [17] and could be associated with a reaction to persisting underlying opportunistic infections such as TB or their residual antigens [14,16,18,19]. This distinct role of T cells in TB and HIV immunology has led to the hypothesis that an unbalanced reconstitution of the T cell compartment contributes to the development of TB-IRIS [20].

Studies of non-pathogen specific IRIS have reported elevated expression of activation markers during IRIS event on either all T cells [9] or exclusively on CD8+ T cells [21] or CD4+ T cells [22]. Although these studies reported no differences in the expression of CD38 and HLA-DR prior to ART, one study reported elevated pre-ART PD-1 expression on CD4+ T cells in IRIS patients [22]. One previous TB-specific IRIS study found no differences in CD8+ or CD4+ T cell activation either before or during ART [23]. Yet in contrast, increased CD8+ T cell activation was recently reported to be specifically relevant during TB-IRIS compared to non-pathogen specific IRIS [24], illustrating the inconsistencies between studies. Although T cell activation is a major driving factor behind T cell maturation, little is known about T cell maturation profiles in TB-specific IRIS. Nevertheless, an unbalanced redistribution during ART of memory T cells with a pro-inflammatory phenotype (e.g. terminally differentiated T cells [25]) could drive IRIS inflammation. A shift from CD8+ and CD4+ central memory T cells to more terminal subtypes has been reported during non-pathogen specific IRIS [21]. However, such shifts have only been sporadically observed elsewhere [22] or not at all [9].

The role of T cell phenotypes in TB-IRIS thus still remains unclear. Importantly, published IRIS studies either did not differentiate between early- and late-onset TB-IRIS or entirely focussed on early-onset TB-IRIS, leaving T cell dynamics in late-onset TB-IRIS largely unexplored. In this study, we therefore compared T-cell activation and maturation markers in early- and late-onset TB-IRIS patients with those in carefully matched controls from a large prospective study in Uganda [6]. Both early- and late-onset TB-IRIS patients showed decreased immune activation prior to ART compared to non-IRIS controls. We also report a maturational shift in late-onset TB-IRIS patients towards more terminal T cell subtypes, which we did not observe in early-onset TB-IRIS.

## Materials and Methods

### Study population

The clinical spectrum of HIV-TB IRIS was studied in a prospective observational study at Mulago Hospital, Kampala, Uganda between 2007–2011 [6,10,26]. HIV-TB co-infected adults treated for active TB infection for less than 2 months and eligible for ART were enrolled in the study. All patients were started on a non-nucleoside reverse transcriptase inhibitor-based ART according to Ugandan national guidelines. The median interval from starting TB-treatment to starting ART was 6 weeks. Patients were followed up for a period of 10 months to monitor paradoxical TB-IRIS development. Sixty (24%) out of 254 HIV-TB co-infected patients developed TB-IRIS. Patients who did not develop IRIS-related symptoms served as non-IRIS controls. Fresh blood samples were collected when patients were diagnosed with inflammatory symptoms consistent with TB-IRIS and at predetermined intervals; before initiation of ART (baseline) and at 1 month, 2 months, 6 months and 9 months after starting ART. In this study, samples taken at baseline and during TB-IRIS or corresponding control time point were analysed. In addition, a group of HIV and TB negative (HIV-TB-) controls was analysed.

### Definitions

*Mycobacterium tuberculosis* infection was diagnosed according to the TB/HIV WHO guidelines [27]. Investigations to confirm TB infection included: clinical examination, chest X-rays and abdominal ultrasounds, sputum smear microscopy for acid-fast bacilli and mycobacterial culture of sputum, aspirate or effusion if available. TB-IRIS cases were classified by a committee of two co-authors (RC and WW) after reviewing all suspected TB-IRIS cases evaluated by the study physicians according to the International Network for the Study of HIV-associated IRIS (INSHI) clinical case-definition [1]. TB-IRIS was diagnosed and sampled when patients presented with at least 1 major criterion (e.g. enlarged lymph nodes) or 2 minor criteria (e.g. fever and cough).

### Patient selection and matching

The INSHI definition of TB-IRIS currently states that patients can be diagnosed with TB-IRIS when symptoms occur within the first 3 months of ART, which includes the majority of TB-IRIS patients [1]. In our cohort, 77% of all TB-IRIS patients developed symptoms within the first month on ART, but additional cases with similar symptoms were diagnosed until 10 months after initiating ART without treatment interruption [6]. To limit TB-IRIS heterogeneity, we classified TB-IRIS patients as early-onset TB-IRIS patients (range 4–28 days on ART) and as late-onset TB-IRIS patients (range 42–307 days on ART). Study eligibility criteria included: adult (>18 years), confirmed HIV-TB infection according to WHO guidelines (when applicable) and eligible for ART. Exclusion criteria included: pregnancy, prior use of ART, Grade 3 renal or liver abnormalities and haemoglobin concentration < 8g/100ml. Patients and controls did not have clinical signs of other opportunistic infections at the time of the study, except for one late-onset TB-IRIS patient who developed genital herpes during ART. None of the TB-IRIS patients or controls were receiving anti-inflammatory treatment when pre-ART or TB-IRIS events were sampled. All TB-IRIS patients were matched 1 by 1 with non-IRIS controls for sex, age (≤ 10 years difference) and baseline CD4 count (+/-15 CD4+ T cells/mm³), these patients were therefore all at a similar stage of HIV-disease progression. The IRIS event from each TB-IRIS patient was paired with the closest available non-IRIS control time point. Early-onset IRIS events were paired with control samples taken at 1 month on ART, while late-onset IRIS events were paired with control samples taken at 1 month (n = 1), 2 months (n = 5), 6 months (n = 1) or 9 months (n = 2) on ART.

### Lymphocyte immunophenotyping

**Fig 1:**
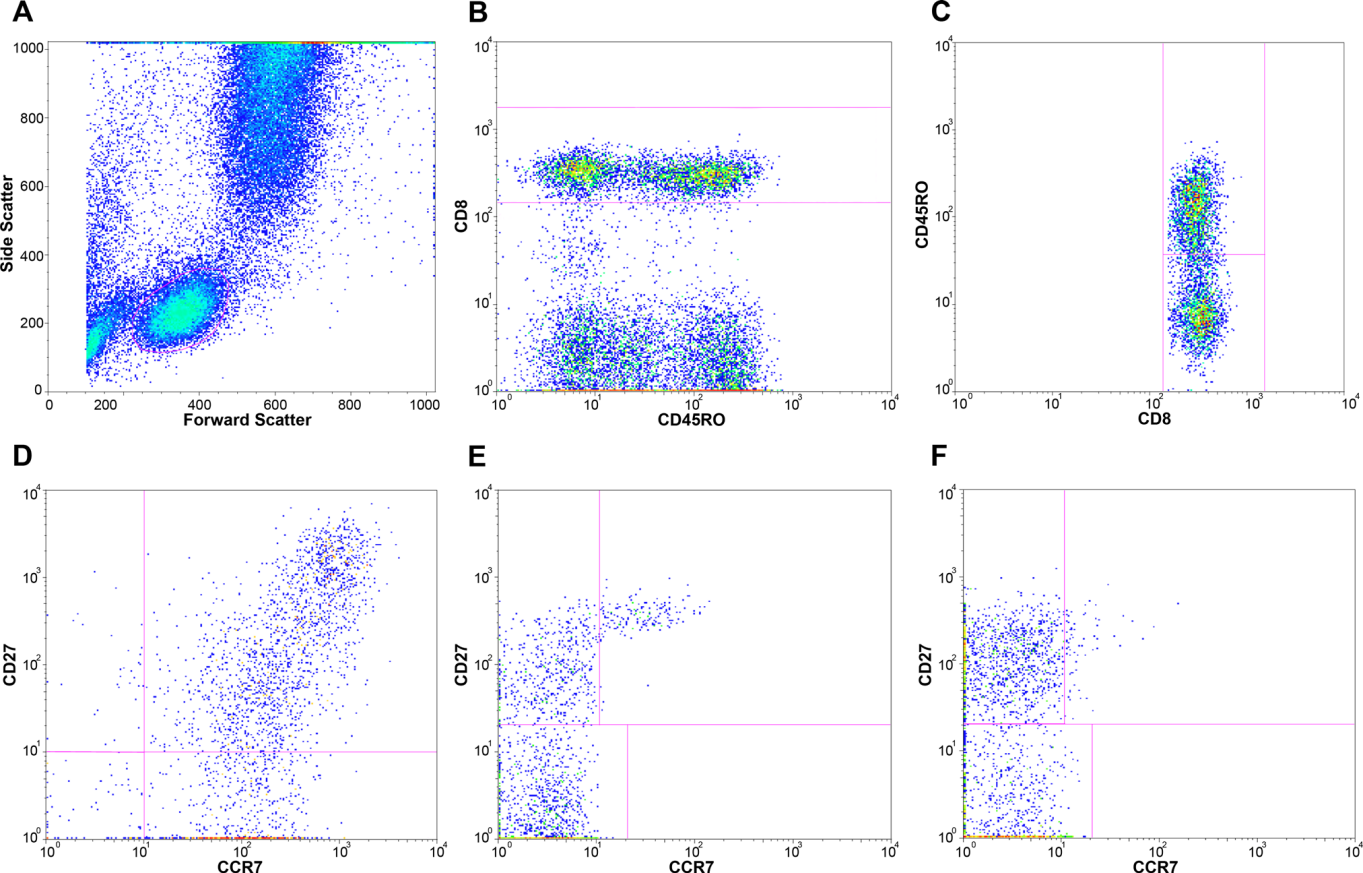
Gating strategy for CD8+ lymphocytes. These plots represent the gating strategy used for flowcytometry analysis of blood samples; A, lymphocytes were gated on a forward scatter area versus side scatter area dotplot; B, CD8+ bright lymphocytes were gated for further determination of subpopulations; C, CD45RO- and CD45RO+ events were gated to determine naïve and memory subpopulations of CD8+ bright lymphocytes; D, activation of memory CD8+ T cells was determined by determining the percentage of CD38 and HLA-DR double positive CD8+ memory lymphocytes. Maturational T cell subpopulations were identified by analysing the (co-)expression of CCR7 and CD27 within E, naïve and F, memory CD8+ bright lymphocytes.

**Fig 2:**
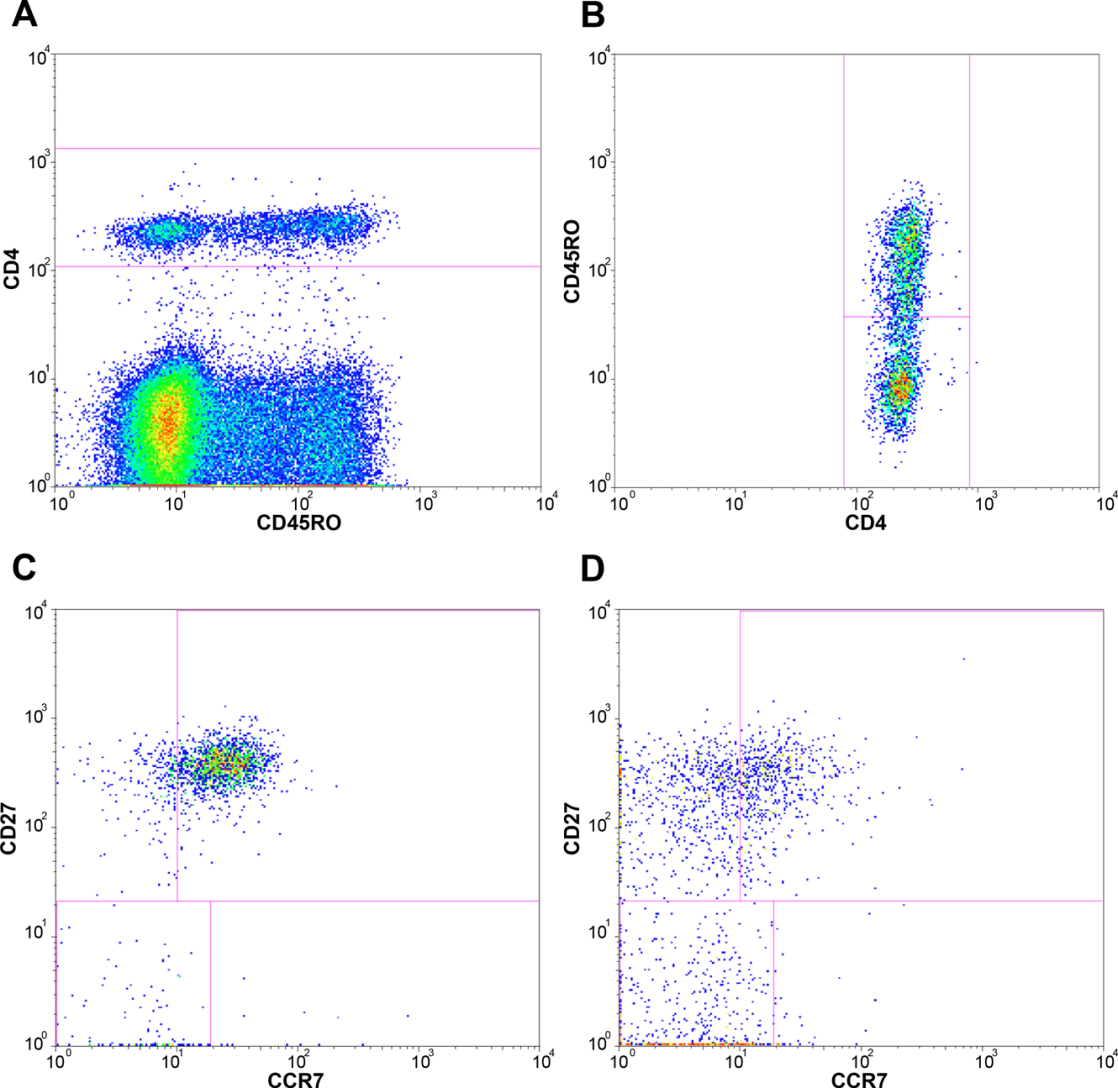
Gating strategy for CD4+ lymphocytes. These plots represent the gating strategy used for flowcytometry analysis of blood samples; lymphocytes were gated on a forward scatter area versus side scatter area dotplot as shown in Fig 1; A, CD4+ bright lymphocytes were gated for further determination of subpopulations; B, CD45RO- and CD45RO+ events were gated to determine naïve and memory subpopulations of CD4+ bright lymphocytes; Maturational T cell subpopulations were identified by analysing the (co-)expression of CCR7 and CD27 within C, naïve and D, memory CD8+ bright lymphocytes.

Fresh whole blood was collected and processed locally within 6 hours of collection. Whole blood was stained with fluorescently labelled antibodies, lysed, washed with PBS and fixed with 1% paraformaldehyde in PBS before measuring with a FACSCalibur four-color flow cytometer (Becton Dickinson (BD)). Three antibody panels were used to determine lymphocyte activation and maturation; panel 1: CD45RO-FITC (Dako), CD38-PE (BD), CD8-PerCP (BD), HLA-DR-APC (BD); panel 2: CD45RO-FITC, CCR7-PE (BD-pharmingen), CD8-PerCP, CD27-APC (eBioscience); panel 3: CD45RO-FITC, CCR7-PE, CD4-PerCP (BD), CD27-APC. Data were analysed with FlowJo software (v9.7 Tree Star), using the following gating strategy. First, lymphocytes were gated on a forward scatter area versus side scatter area dotplot (Fig 1). Next, CD4+ bright and CD8+ bright lymphocytes were gated for further determination of subpopulations. Activation of memory CD8+ T cells (panel 1) was determined by gating CD8+ bright lymphocytes for CD45RO+ events and subsequently determining the percentage of CD38 and HLA-DR double positive CD8+ memory lymphocytes (Fig 1). Maturational T cell subpopulations were identified by gating CD8+ bright (panel 2) or CD4+ bright (panel 3) lymphocytes for CD45RO+ and CD45RO- events and subsequently analysing the (co-)expression of CCR7 and CD27, as described previously (Figs 1 and 2) [28,29]. Analysed subpopulations were expressed as frequencies of total CD8+ bright or CD4+ bright lymphocytes and included: naïve (Tn; CD45RO-CCR7+CD27+), central memory (Tcm; CD45RO+CCR7+CD27+), effector memory (Tem; CD45RO+CCR7-CD27+), terminal effector memory (Ttem; CD45RO+CCR7-CD27-), early effector (Tearly eff; CD45RO-CCR7-CD27+) and effector (Teff; CD45RO-CCR7-CD27-) T cells.

### Ethical considerations

The study was approved by: the Research Committee of the Infectious Diseases Institute (IDI), the ethical review board of Makerere University, the Uganda National Council of Science and Technology, the institutional review board of the Institute of Tropical Medicine of Antwerp and the Ethics Committees of the Faculties of Medicine of the University of Antwerp. Written informed consent was obtained from all study participants.

### Statistical analysis

Paired comparisons between TB-IRIS patients and non-IRIS controls were done using the Wilcoxon signed-rank test. Unpaired comparisons between early- and late-onset TB-IRIS patients and with HIV-TB- controls were done using the Mann-Whitney U test. Statistics were performed using SPSS software (version 17.0), GraphPad Prism (version 5) or R (version 2.15.3) with significance level set at p < 0.05. Because of the hypothesis driven nature of this study, no correction for multiple testing was applied [30,31].

## Results

### Study population

Twenty-two early-onset TB-IRIS patients were selected, of whom 18 had flow cytometry data available prior to ART initiation and 16 had data available during IRIS event. In addition, 10 late-onset TB-IRIS patients were selected, of whom 8 had data available prior to ART initiation and 9 had data available during IRIS event. TB-IRIS patients were paired at each time point with an equal number of non-IRIS controls. Prior to treatment, 14/22 early-onset TB-IRIS patients and 13/22 non-IRIS controls had pulmonary TB, while 8/22 early-onset TB-IRIS patients and 9/22 controls had extrapulmonary TB. For both late-onset TB-IRIS patients and controls, 8/10 had pulmonary TB and 2/10 had extrapulmonary TB. Early-onset TB-IRIS patients and their non-IRIS controls did not differ for age, sex, baseline CD4 count, baseline viral load or TB treatment duration prior to ART (Table 1). Baseline characteristics of late-onset TB-IRIS patients and non-IRIS controls were also similar, except for age. Though within matching criteria, late-onset TB-IRIS patients were a median of 5 years older than their non-IRIS controls (p = 0.028). No differences in baseline characteristics were observed between early-onset and late-onset TB-IRIS patients. Early-onset TB-IRIS occurred a median 15 (14–27) days after starting ART, compared to 98 (60–196) days for late-onset TB-IRIS patients (p < 0.001). Compared to their matched non-IRIS control time points, early-onset TB-IRIS events occurred a median of 14 days (interquartile range (IQR): 4–16) earlier (p = 0.002) during ART. Late-onset TB-IRIS events occurred a median of 29 (IQR: 10–55) days later (p = 0.022) during ART than their respective non-IRIS control time points. An additional group of 16 HIV and TB negative subjects (HIV-TB- controls) was also analysed, who did not differ significantly for age or sex from TB-IRIS patients (data not shown).

**Table 1:** Characteristics of TB-IRIS patients and matched controls for flow cytometry.

	Early TB-IRIS			Late TB-IRIS			Early vs. Late
Variables	TB-IRIS (n = 22)	Controls (n = 22)	P^*a*^	TB-IRIS (n = 10)	Controls (n = 10)	P^*a*^	P^*b*^
**Characteristics Baseline**							
Sex male n (%)^*c*^	12 (55)	12 (55)	1.000	7 (70)	7 (70)	1.000	0.409
Age (years)	40 (34–43)	39 (35–43)	0.822	40 (36–49)	35 (33–42)	**0.028**	0.568
CD4 (cell/mm³)	25 (12–59)	30 (17–61)	0.398	51 (21–128)	50 (21–111)	0.343	0.230
TB treatment prior to ART (days)	42 (25–63)	37 (25–60)	0.394	55 (28–73)	40 (29–51)	0.285	0.360
Viral Load (Log copies/ml)^*d*^	5.7 (5.3–5.7)	5.6 (5.2–5.8)	0.918	5.3 (2.9–6.9)	5.5 (5.2–5.7)	0.593	0.688
**Characteristics TB-IRIS event** ^*e*^							
Days since start of ART ^*f*^	15 (14–27)	29 (28–30)	**0.002**	98 (60–196)	56 (54–152)	**0.022**	**<0.001**

Values are shown as median values with interquartile range. Median age difference in years between late-onset TB-IRIS patients and controls was 5 (IQR: 1.75–7.5).

^a^Wilcoxon signed-rank test.

^b^Mann-Whitney U test.

^c^Chi square fishers exact test for binominal data.

^d^For early TB-IRIS cases and controls; n = 16, viral loads were only available for 3 late-onset TB-IRIS patients.

^e^For early TB-IRIS cases and controls; n = 16, for late TB-IRIS cases and controls; n = 9.

^**f**^Median time difference between IRIS event and corresponding control time point was 14 (4–16) days for early-onset TB-IRIS and 29 (10–55) days for late-onset TB-IRIS.

### Decreased immune activation in early- and late-onset TB-IRIS prior to ART

**Fig 3:**
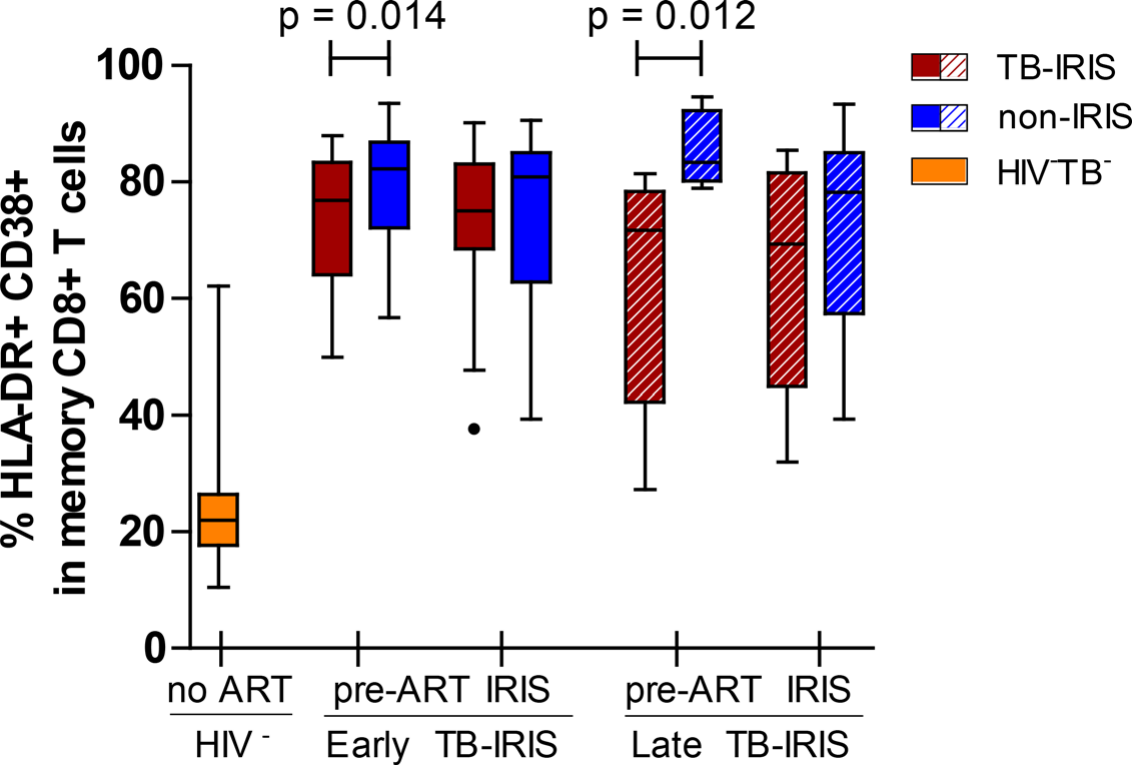
Percentage of activated CD8+ cells in early- and late-onset TB-IRIS patients. This box and Tukey whisker plot represents median percentages and IQR of HLA-DR+/CD38+ cells within CD8+/CD45RO+ T cells for early- and late-onset TB-IRIS patients (red) compared to non-IRIS (blue) controls. Median values and IQR for 16 HIV-TB- (yellow) controls are also represented. Full lines above indicate significant differences between paired patients (Wilcoxon signed-rank test). The level of significance was set to P < 0.05 for all tests. Number of patients (and paired non-IRIS controls) prior to ART were; 18 for early-onset TB-IRIS and 8 for late-onset TB-IRIS. Number of patients (and paired non-IRIS controls) during IRIS event or corresponding control time point were; 16 for early-onset TB-IRIS and 9 for late-onset TB-IRIS.

To assess the putative role of an overly-activated T cell compartment in TB-IRIS, the percentage of activated (HLA-DR+/CD38+) memory CD8+ T lymphocytes in fresh peripheral whole blood samples was compared between TB-IRIS patients and non-IRIS controls (Fig 3). We observed a lower percentage of activated CD8+ T cells in early-onset TB-IRIS patients prior to ART (77% vs. 82%, p = 0.014) compared to non-IRIS controls, but no differences during IRIS event. Similarly, we observed a lower percentage of activated CD8+ T cells prior to ART in late-onset TB-IRIS patients compared to non-IRIS controls (71% vs. 83%, p = 0.012), but no differences during IRIS event. As expected, both early- and late-onset TB-IRIS patients showed significantly elevated percentages of activated CD8+ T cells compared to HIV-TB- controls at any given time point (p ≤ 0.001).

### Memory-effector CD8+ T cell shift during late-onset but not early-onset TB-IRIS

**Fig 4:**
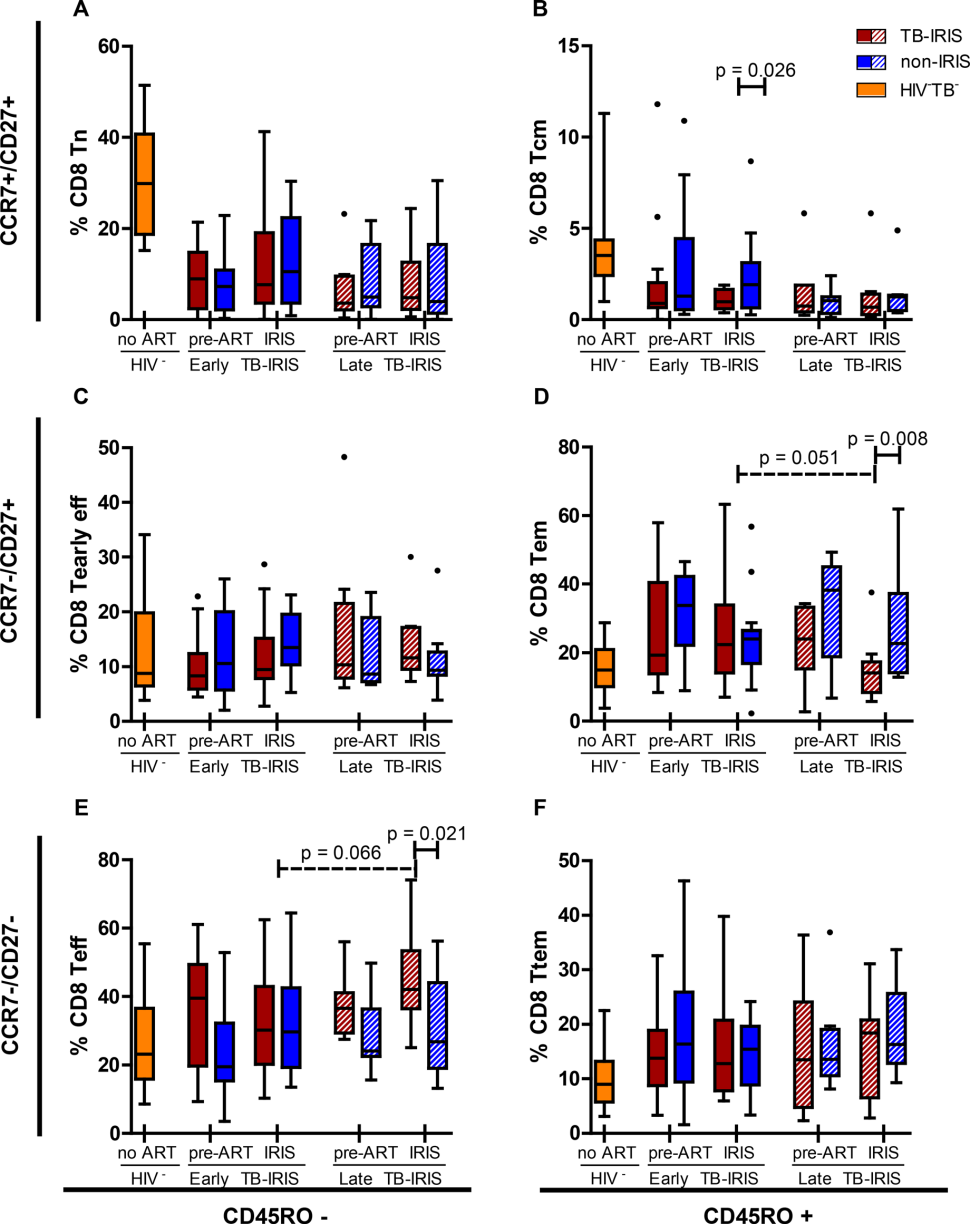
Percentage of CD8+ maturation sub-stages in early-onset TB-IRIS patients. These box and Tukey whisker plots represent median percentages and IQR of; A, naïve cells (Tn); B, central memory cells (Tcm); C, early effector cells (Tearly eff); D effector memory cells (Tem); E, effector cells (Teff); F, terminal effector memory cells (Ttem) within CD8+ T cells for early- and late-onset TB-IRIS patients (red) and non-IRIS controls (blue), 16 HIV-TB- (yellow) controls. Full lines above indicate significant differences between paired patients (Wilcoxon signed-rank test). Dashed lines above indicate significant differences between unpaired patient groups (Mann-Whitney U test). The level of significance was set to P < 0.05 for all tests. Number of patients (and paired non-IRIS controls) prior to ART were; 17 for early-onset TB-IRIS and 8 for late-onset TB-IRIS. Number of patients (and paired non-IRIS controls) during IRIS event or corresponding non-IRIS control time point were; 16 for early-onset TB-IRIS and 9 for late-onset TB-IRIS.

To investigate whether TB-IRIS is associated with maturation abnormalities of the CD8+ T cell subset, maturation stages of CD8+ T cells were studied in peripheral blood of TB-IRIS patients and controls (Fig 4). Based on the expression of CD45RO, CCR7 and CD27, T cell subsets were subdivided in naïve (Tn), central memory (Tcm), effector memory (Tem), terminal effector memory (Ttem), early effector (Tearly eff) and effector (Teff) T cells. Prior to ART, no differences were observed between early- or late-onset IRIS patients and their non-IRIS controls. During IRIS event, early-onset TB-IRIS patients showed a slightly lower percentage of CD8+ Tcm cells compared to non-IRIS controls (1.0% vs. 1.9%, p = 0.026). At this time point, late-onset TB-IRIS patients showed markedly lower percentages of CD8+ Tem cells (14% vs. 23%, p = 0.008) and markedly higher percentages of CD8+ Teff cells (42% vs. 27%, p = 0.021) compared to non-IRIS controls. This shift was not observed in early-onset TB-IRIS patients. In fact, late-onset IRIS events showed trends towards lower CD8+ Tem (p = 0.051) and higher CD8+ Teff (p = 0.066) frequencies compared to early-onset IRIS events. Both early- and late-onset TB-IRIS patients showed significantly lower percentages of CD8+ Tn and Tcm subsets compared to HIV-TB- controls at both time points (p ≤ 0.031).

### Memory-effector CD4+ T cell shift during late-onset but not early-onset TB-IRIS

**Fig 5:**
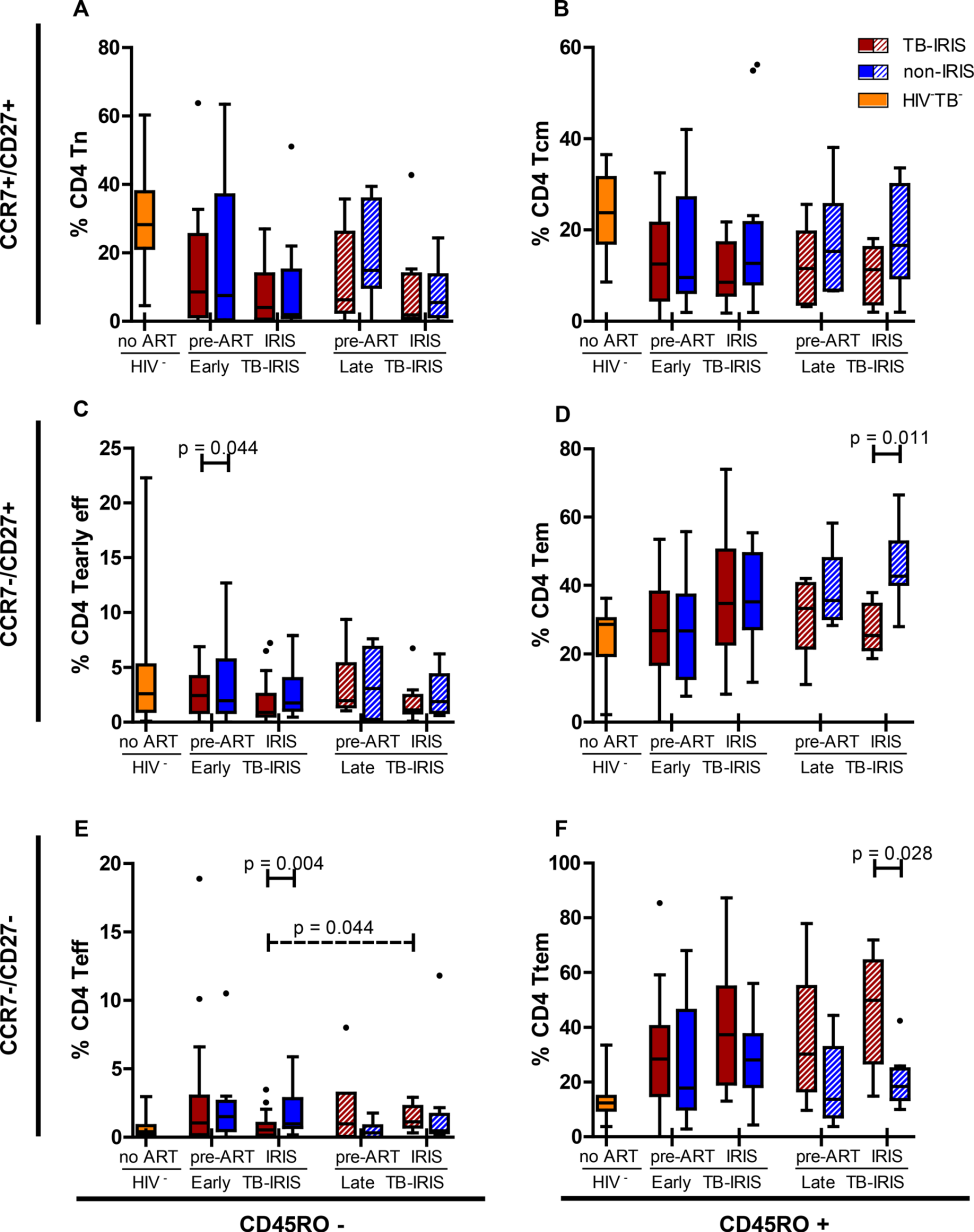
Percentage of CD4+ maturation sub-stages in early-onset TB-IRIS patients. These box and Tukey whisker plots represent median percentages and IQR of; A, naïve cells (Tn); B, central memory cells (Tcm); C, early effector cells (Tearly eff); D effector memory cells (Tem); E, effector cells (Teff); F, terminal effector memory cells (Ttem) within CD4+ T cells for early- and late-onset TB-IRIS patients (red) and non-IRIS controls (blue), 16 HIV-TB- (yellow) and 11 HIV-TB+ (green) controls. Full lines above indicate significant differences between paired patients (Wilcoxon signed-rank test). Dashed lines above indicate significant differences between unpaired patient groups (Mann-Whitney U test). The level of significance was set to P < 0.05. Number of patients (and paired non-IRIS controls) prior to ART were; 17 for early-onset TB-IRIS and 6 for late-onset TB-IRIS. Number of patients (and paired non-IRIS controls) during IRIS event or corresponding non-IRIS control time point were; 16 for early-onset TB-IRIS and 9 for late-onset TB-IRIS.

Finally, we explored the distribution of maturation stages (Tn, Tcm, Tem, Ttem, Tearly eff and Teff) in the CD4+ T cell compartment (Fig 5). Similar to the CD8+ T cell compartment, we observed no differences pre-ART. During early-onset IRIS event, we observed slightly lower proportions of CD4+ Tearly eff cells (0.9% vs. 1.8%, p = 0.044) and Teff cells (0.6% vs. 1.0%, p = 0.004) compared to non-IRIS controls. Proportions of CD4+ Teff cells at early-onset IRIS event were also lower compared to those at late-onset IRIS event (p = 0.044). In contrast, late-onset IRIS events again showed a shift in maturation steps: proportions of CD4+ Tem cells were lower (25% vs. 43%, p = 0.011) and proportions of Ttem cells were higher (27% vs. 14%, p = 0.028) compared to non-IRIS controls. Both early- and late-onset TB-IRIS patients showed significantly lower percentages of CD4+ Tn and Tcm cells compared to HIV-TB- controls at both time points (p ≤ 0.027).

## Discussion

HIV-TB patients with low CD4 counts who start ART are at high risk of developing TB-IRIS [6,32]. Although the immunopathogenesis of TB-IRIS is still not completely understood, the explosive restoration of T cell function is believed to play a distinct role [20–22,33]. In the present study, we compared T cell activation and maturation in fresh whole blood samples between Ugandan TB-IRIS patients and matched controls before ART initiation and at IRIS event. Approximately 75% of TB-IRIS patients in our cohort developed TB-IRIS early (< 1 month) during ART [6]. Approximately 25% of TB-IRIS patients developed symptoms at later intervals (> 1 month) during ART, although with otherwise similar clinical symptoms. Since late-onset TB-IRIS has never been studied as a separate group, we decided to compare late-onset TB-IRIS with early-onset TB-IRIS. Our data show lower CD8+ T cell activation levels prior to ART initiation in both early- and late-onset TB-IRIS patients compared to non-IRIS controls. During IRIS event, however, the observed difference in T cell activation disappeared. Instead, late-onset but not early-onset TB-IRIS patients developed a shift towards terminal effector T cell subpopulations when TB-IRIS occurred.

We report lower levels of T cell activation in both early-onset and late-onset TB-IRIS patients prior to ART, suggesting common pre-ART mechanisms leading to early- and late-onset TB-IRIS. On one hand, such mechanisms could involve a lowered cytotoxic function as well as reduced local production of interferon-gamma by the CD8 T cell compartment. However, previous studies [23,34] as well as our own findings [35] suggest that interferon-gamma responses to TB-antigens are similar between TB-IRIS patients and controls prior to ART. On the other hand, we previously observed lower pre-ART IL-6 and lipopolysaccharide-binding protein levels in plasma from TB-IRIS patients from our cohort [36], which is in line with the lower level of CD8 T cell activation observed here. We believe that these lower cytokine levels reflect the inability of the innate immune system to mount an effective response to the pre-ART TB antigen load. T cell activation is dependent on antigen presenting cells [37] and IL-6 has been shown to induce CD8 T cell activation [38,39]. Therefore we hypothesize that the lower pre-ART CD8 T cell activation levels in TB-IRIS patients could be a downstream consequence of this diminished innate response, rather than a sign of diminished CD8+ T cell function. Interestingly, it was previously suggested that an impaired innate ability to respond to the pre-ART antigen load could lead to priming of the innate immune system, followed by an inflammatory burst when ART is initiated [40]. Our data thereby provide further evidence of an impaired immune response prior to ART leading to early- and late-onset TB-IRIS. During TB-IRIS event, the observed difference in T cell activation disappeared and we did not observe elevated T cell activation, in contrast to previous studies [21,22,33]. Interestingly, we have previously shown that the cytokine storm during TB-IRIS is dominated by innate factors [36]. Moreover, the causal role of excessive T cell responses in TB-IRIS has previously been questioned [23]. Although it is not clear why CD8+ T cell activation did not rise in parallel with this cytokine storm, our data thus do not support the presence of an over-activated CD8+ T cell compartment in TB-IRIS patients.

Persistent immune activation during HIV infection typically coincides with a depletion of the naïve T cell pool [41–43]. However, little is known about T cell maturation profiles in TB-specific IRIS. We observed slightly lower percentages of CD8+ Tcm cells during early IRIS-event and a subtle decrease in CD4+ effector populations. One possible explanation would be that these subsets migrated to tissue in response to the local inflammation during early-IRIS event. In contrast to early-onset IRIS, late-onset IRIS was characterized by a much more pronounced shift from memory to effector T cell subpopulations, resembling the one observed in a previous non-pathogen specific IRIS study [21]. This study had a large proportion (41%) of TB-IRIS cases and reported a shift from CD8+ and CD4+ central memory T cells to a more terminally differentiated subtype during IRIS. The median time to IRIS was 38 (IQR, 24–56) days on ART, a time frame in between that of our early- and late-onset TB-IRIS patients. The phenotypic maturation of T cells is believed to be dependent on antigen-load and cytokine environment [44–46]. Since TB-IRIS is associated with a high antigen load and a cytokine storm [36,47,48], one could argue that the exposure to this inflammatory environment induces a maturational shift during the redistribution of the T cell compartment. A longer period on ART allows for a greater redistribution of the memory T cell pool and possibly a longer exposure to this environment. It is therefore tempting to speculate that for these reasons, the maturational shift was more pronounced in patients who developed TB-IRIS at a later time during ART. Future studies should investigate the possible involvement of persisting antigen loads and elevated cytokine environments in late-onset TB-IRIS development.

Since all our TB-IRIS patients were closely matched to non-IRIS controls for CD4 count and viral load, we do not expect a bias due to differences in disease stage. However, the unpredictability of TB-IRIS poses a serious challenge for prospective studies to adequately match control time points to IRIS events. As a result, early control time points were a median of 2 weeks later than their matched early-onset TB-IRIS patients. Since this bias probably underestimated residual T cell activation in controls, it likely has not affected our conclusion of a lack of increased T cell activation at early-onset TB-IRIS. Late control time points were a median of 1 month earlier than late-onset TB-IRIS cases. This bias probably overestimated terminal effector T cell populations in controls, thus actually underestimating the observed shift to terminal subtypes in late-onset TB-IRIS patients. Nevertheless, given the small number of early- and late-onset TB-IRIS patients and the relatively large age difference between late-onset TB-IRIS patients and controls, further studies differentiating between early- and late-onset TB-IRIS are required to confirm our findings.

Taken together, our data provide evidence of reduced CD8+ T cell activation prior to ART leading to early- and late-onset TB-IRIS and do not suggest the presence of an over-activated CD8+ T cell compartment at TB-IRIS event. In addition, we provide the first indications of heterogeneous T cell maturation between early-onset and late-onset TB-IRIS, with late-onset TB-IRIS patients experiencing a more terminally differentiated maturation profile. Our study hereby shows that early- and late-onset TB-IRIS may share common predisposing factors, yet appear to be set apart by a different pathogenesis at the time of the disease. These presentations of TB-IRIS should therefore be studied separately in the future. A better understanding of the immune responses before and during ART in TB-IRIS patients could lead to novel markers for the detection and prevention of this important complication.
